# Quality of Life and Work Productivity Impairment of Patients with Allergic Occupational Rhinitis

**Published:** 2019-01

**Authors:** Maher Maoua, Olfa El Maalel, Imène Kacem, Sana Guedri, Maha Ben Kacem, Sana Aissa, Monia Ghammem, Aicha Brahem, Houda Kalboussi, Faten Debbabi, Souhaiel Chatti, Néjib Mrizak

**Affiliations:** 1 Department of Occupational Medicine – Teaching Hospital Farhat Hached, Avenue Ibn El Jazzar, Sousse, 4000, Tunisia; 2 University of Sousse, Faculty Of Medicine Ibn El Jazzar, Avenue Mohamed Karoui, 4002, Sousse, Tunisia; 3 Department of Pneumology and allergology – Teaching Hospital Farhat Hached, Avenue Ibn El Jazzar, Sousse, 4000, Tunisia; 4 Department of Otorhinolaryngology – Teaching Hospital Farhat Hached, Avenue Ibn El Jazzar, Sousse, 4000, Tunisia

**Keywords:** Allergic occupational rhinitis, Quality of life, Productivity, Impairment

## Abstract

**Background:**

Several studies demonstrated the negative impact of allergic rhinitis on Quality of Life (QOL) and occupational activities. Similar studies on allergic Occupational Rhinitis (OR) are rare. The aim of this study was to evaluate the QOL and work productivity of patients diagnosed with allergic occupational rhinitis.

**Materials and Methods:**

We conducted a cross-sectional study from January 2005 to December 2015 at the Department of Occupational Medicine in Farhat Hached Teaching Hospital-Tunisia including patients diagnosed with allergic OR. QOL was assessed by the Mini-RQLQ (Rhinitis quality of life questionnaire) and Work impairment was measured by WPAI (Work Productivity and Activity Impairment) questionnaire.

**Results:**

a total of 414 patients was enrolled in the study with a mean age of 37.82±8.08 years and a sex ratio = 0.33. Textile and clothing industry was the most represented sector (65.7%). The mean percent work time missed (absenteeism) due to allergic OR was 9.98±20.86% with a median of 0% and the mean presenteeism score was 46.7±32.67%. Overall QOL was 2.71±1.31. The most affected domains were practical problems and activity limitations. Absenteeism was positively correlated with age and eye symptoms scores. Both presenteeism and percent overall activity impairment were positively correlated with severe nasal obstruction and activity limitations score.

**Conclusion:**

Allergic OR impairs QOL and work productivity. Although it doesn’t seem to be associated with an important absenteeism, work productivity is reduced by an important rate of presenteeism. QOL and work productivity seem to interact significantly.

## INTRODUCTION

Rhinitis is defined as an inflammation of nasal mucosa, characterized by nasal symptoms such as nasal airflow limitation, anterior and/or posterior rhinorrhea, nasal pruritus and sneezing (1).

Occupational Rhinitis (OR) is an inflammatory disease of the nose, which is characterized by intermittent or persistent symptoms (i.e., nasal congestion, sneezing, rhinorrhea, itching), and/or variable nasal airflow limitation and/or hypersecretion arising out of causes and conditions attributable to a particular work environment and not to stimuli encountered outside the workplace (2). Work-related rhinitis may be distinguished from work-exacerbated rhinitis that is pre-existing or concurrent rhinitis exacerbated by workplace exposures (2). OR can be classified into allergic and non-allergic. The second type of OR is caused by irritants and non-immunological reactions (2).

Occupational allergic rhinitis is habitually the inaugural clinical expression of airways sensitization to an allergen existing in the work environment (3).

Allergic rhinitis affects 10 to 25% of the population in developed countries and its prevalence is growing in developing countries (4–7). Its prevalence is about 15% in Switzerland and 20% in Europe (8). Epidemiological data available in Switzerland since 1926 have shown an increasing amount of sensitization to pollens from 1 to 12% within 70 years (8). The presumed causes of the increase in prevalence are multifactorial including air pollution, ozone, urbanization tendency, western lifestyle, stress, etc. (8).

Several studies showed that 15% of workers have OR which represents 4% of occupational respiratory diseases (9). Type and reactivity of allergens, work conditions, type of industrial techniques and processes, age and atopy are examples of risk factors that have an effect on the prevalence of OR (10).

The incidence and prevalence of occupational allergic rhinitis are unknown in Tunisia. Nevertheless, many studies were conducted in some specific sectors. Data from the Tunisian National Health Insurance Fund (CNAM) show a small number of declared cases underlining an under-declaration (10).

The evolution of Allergic OR to asthma is well-known and rhinitis is often preceding asthmatic symptoms (11). However, OR is 2 to 3 times more frequent than occupational asthma (12).

Allergic rhinitis is a public health concern due to its prevalence, costs, association with asthma and negative impact on Quality of Life (QOL) and work abilities (13).

Studies demonstrating the deterioration of QOL (14–19) and occupational productivity (14,18, 20) in patients with allergic rhinitis are numerous, but such studies are rarer for allergic OR.

Therefore, we conducted this study with the objectives of evaluating QOL and productivity of patients with allergic occupational rhinitis.

## MATERIALS AND METHODS

### Study design

We conducted a cross-sectional study during the period from January 2005 to December 2015 at the Department of Occupational Medicine in Farhat Hached Teaching Hospital-Tunisia. Patients aged 18 to 65 years, diagnosed with allergic occupational rhinitis after clinical and paraclinical investigations, according to the diagnostic algorithm of the European Academy of Allergy and Clinical Immunology (EAACI) (2), were enrolled in the study. Patients diagnosed with non-allergic OR and those with work-exacerbated rhinitis were excluded. Due to frequent association between OR and occupational asthma and its impact on the evaluation of QOL, patients were explored by spirometry and non-specific bronchoprovocation. Asthmatic patients were also excluded from the study.

Data collected from patients’ medical records were age, gender, matrimonial status, extraprofessional activities, job station, professional qualifications, products handled at work, sector of activity, use of personal protective equipment, occupational seniority, personal and familial medical history, physical examination findings and results of paraclinical examinations.

### Measurement tools

QOL was measured by the Arabic validated version of the Mini-Rhinoconjunctivitis Quality of Life Questionnaire (MiniRQLQ) exploring five domains: activities, practical problems, nose symptoms, eye symptoms and other symptoms. The original version of the questionnaire was developed by Juniper EF et al. in 1991 (21,22) and included 28 items. The Arabic version was validated by AbuRuz et al*.* in 2009 (23). Like the RQLQ, all items of the mini-RQLQ are equally weighted and the analysis is conducted exactly the same manner as for the RQLQ.

Patients were invited to answer each question using a scale of seven points (0=not troubled, 6=extremely troubled). Items of each domain were added to reproduce scores from 0 to 6 where 0 is the best QOL and 6 is the worst possible QOL score. The questionnaire is analyzed directly from the scores recorded and the results are expressed as the mean score per item for each of the domains as well as for overall QOL. Overall QOL score is estimated from the mean score of all the items.

Work Productivity and Activity Impairment questionnaire (WPAI-AS) (24,25) was used to evaluate limitations of occupational activities secondary to allergic OR. The WPAI yields four types of scores: Absenteeism (work time missed), presenteeism (impairment at work/reduced on-the-job effectiveness), work productivity loss (overall work impairment/absenteeism plus presenteeism) and activity impairment. WPAI outcomes are expressed as impairment percentages, with higher numbers indicating greater impairment and less productivity.

### Statistical analysis

Means, standard deviations, medians and extreme values were calculated for quantitative variables. Frequencies and percentages were calculated for qualitative variables.

Pearson correlation coefficient was used to explore the correlation between two quantitative variables. Student’s t-test for the comparison of two means and Snedecor’s F test for the comparison of several means were performed. Multiple linear regressions were conducted for multivariate analysis. For all statistical tests, the threshold of significance (P-value) was set to 0.05.

## RESULTS

### Sociodemographic and medical characteristics

During the study period, 414 patients were diagnosed with allergic occupational rhinitis. The mean age of the population was 37.82±8.08 years with extremes from 19 to 65 years. A female predominance was noted with 311 women (75.1%) versus 103 men and a sex ration of 0.33.

Among the study population, 395 workers were unskilled laborers (95.4%), 7 were skilled laborers (1.7%) and only 12 patients were highly qualified (2.9%). Table 1 shows occupational sectors of the cases with a clear predominance of the clothing and textile sector.

**Table 1.** Distribution of population by gender and sector of activity

**Sector of activity**	**Gender**		**Total**	

	Men	Women	N	%
**Clothing and textile**	29	243	272	65.7
**Wood industry**	11	1	12	2.9
**Food industry**	20	1	21	5.1
**Chemical industry**	13	9	22	5.3
**Electronics industry**	5	19	24	5.8
**Health sector**	1	10	11	2.7
**Hotels**	2	11	13	3.1
**Construction sector**	3	1	4	1
**Metallurgical industry**	4	0	4	1
**Plastic industry**	1	2	3	0.7
**Gardening**	2	0	2	0.5
**Other sectors**	12	14	26	6.3
**Total**	103	311	414	100

Although exposed to various dusts, vapors and fumes, only 10 patients usually wore protective masks during their activity (2.4%). Average professional seniority was 15.34 ± 8.84 years and 196 workers (47.3%) had a seniority more than 15 years.

Only 32 patients were smokers (7.7%) and six patients were exposed to passive smoking (1.4%).

The delay between the first occupational exposure and the onset of rhinitis ranged from 1 month to 32 years with an average of 9.84 ± 7.69 years.

Table 2 resumes complaints from patients, clinical examination findings and results of rhinomanometry.

**Table 2.** Complaints, physical examination findings and rhinomanometry results

	**N**	**%**
**Complaints**		
Sneezing	308	74.4
Itching	306	73.9
Nasal obstruction	294	71
Rhinorrhea	267	64.5
Anosmia	20	4.8
**Physical examination findings**		
Pale nasal mucosa	52	12.6
Nasal polyposis	8	0.2
hypertrophy of the middle and inferior nasal turbinates	4	1
**Rhinomanometry**	355	85.7
Normal	26	7.3
Bilateral obstruction	317	89.3
Severe	205	57.7
Moderate	78	22
Mild	34	9.6
Unilateral obstruction	12	3.4
Severe	1	0.3
Moderate	5	1.4
Mild	6	1.7

The most frequent etiological suspected agent was cotton dust for 287 patients (69.3%) who declared the onset of symptoms during exposition. Detergents (5.1%), colophony (4.3%), cereals and flour (4.1%) were other frequent suspected etiologies (Table 3).

**Table 3.** Suspected causative agents of allergic occupational rhinitis

**Suspected Causative agents**	**Number of cases**	**Percentage**
**Cotton dust**	287	69.3
**Detergents**	21	5.1
**Colophony**	18	4.3
**Cereals and flour**	17	4.1
**Glues**	14	3.4
**Paints and varnishes**	13	3.1
**Wood dust**	12	2.9
**Epoxy resin**	10	2.4
**Formaldehyde**	7	1.7
**Metal dust**	5	1.2
**Plastics**	4	1
**Leather dust**	3	0.7
**Solvents**	3	0.7
**Total**	414	100

### QOL and work productivity and activity impairment:

Overall QOL score was 2.71±1.31. The most affected domains were practical problems and activity limitations (Table 4).

**Table 4.** Mean scores of the Mini-RQLQ domains among patients with allergic OR

**Domains**	**Mean score**	**SD**
**Activity limitations**	3.02	± 1.74
Regular activities	3.41	± 1.98
Recreational activities	2.31	± 1.96
sleep	3.34	± 2.20
**Practical problems**	3.48	± 2.10
Need to rub nose/eyes	3.34	± 2.37
Need to blow nose repeatedly	3.63	± 2.48
**Nose symptoms**	2.9	± 1.72
sneezing	3.41	± 2.30
Stuffy blocked nose	2.77	± 2.37
Runny nose	2.52	± 2.37
**Eye symptoms**	1.36	± 1.57
Itchy eye	1.81	± 2.31
Sore eyes	1.26	± 2.05
Watery eyes	1.01	± 1.88
**Other symptoms**	2.81	± 1.62
Tiredness and/or fatigue	3.46	± 2.24
Thirst	1.68	± 2.00
Feeling irritable	3.29	± 2.20
Overall quality of life score	2.71	± 1.31

In the week prior to the survey, the mean percent work time missed (absenteeism) due to allergic occupational rhinitis was 9.98 ±20.86% with a median of 0% indicating that half of patients weren’t absent during the last week because of their disease.

However, presenteeism was much higher with a mean percent impairment while working due to allergic OR of 46.7±32.67% during the last week and a median score of 60%. Percent overall work impairment was 48.88% ± 34.5% and percent activity impairment was 44.71± 35.41%.

Absenteeism was correlated with age of patients with P=0.015 and r=0.26 indicating higher absenteeism rates in older workers. This indicator wasn’t associated with gender, sector of activity, occupational seniority number of symptoms or to rhinomanometry findings. Presenteeism wasn’t statistically associated with any sociodemographic or medical variable.

Percent overall work impairment was positively correlated with age (P=0.045; r=0.22) while percent activity impairment was higher among women (P=0.001), workers in the clothing and textile sector (P=0.026), patients with bilateral obstruction (P=0.008), patients with severe nasal obstruction on rhinomanometry (P=0.016) and was positively correlated with age (P=0.037; r=0.18) and professional seniority (P=0.006; r=0.23).

Analysis of Mini-RQLQ items showed that activity limitations score was higher among textile workers (P=0.009), among patients with bilateral (P=0.001) and severe (0.007) nasal obstruction and those with higher professional seniority (P=0.022; r=0.19).

Female workers had higher practical problems (P=0.04), eye symptoms (P=0.02), other symptoms (P=0.009) and overall QOL (P=0.01) scores.

All domains of the Mini-RQLQ were positively correlated with absenteeism, presenteeism, work productivity loss and activity Impairment with higher WPAI percentages associated with worst QOL scores. Correlations between overall QOL score and WPAI indicators showed P-values ≤10^−3^ and r values at 0.4 for absenteeism, 0.58 for presenteeism, 0.61 for percent overall work impairment and 0.53 for activity impairment.

Multiple linear regressions were performed with scores of absenteeism, presenteeism, work productivity loss and activity impairment as dependent variables. For each regression, statistical models included gender, age, professional seniority, sector of activity, Mini-RQLQ scores and rhinomanometry results. Absenteeism was positively correlated with age of patients and eye symptoms scores indicating that older patients with higher eye symptoms scores on Mini-RQLQ had greater absence rates. Percent activity impairment was higher among women and patients with higher activity limitations scores. Both presenteeism and percent overall activity impairment were positively correlated with severe nasal obstruction on rhinomanometry and activity limitations score (Table 5).

**Table 5.** Results of multiple linear analysis between WPAI scores and associated variables

**WPAI scores**	**Associated variables**	**p-value**	**Beta coefficient**
**Absenteeism**	Age	0.005	0.32
	Eye symptoms	0.002	0.35
**Presenteeism**	Sever nasal obstruction	0.012	0.24
	Activity limitations score	<10^−3^	0.7
**Work productivity loss**	Sever nasal obstruction	0.016	0.23
	Activity limitations score	<10^−3^	0.73
**Activity impairment**	Gender	0.001	0.2
	Activity limitations score	<10^−3^	0.73

## DISCUSSION

This study is, to our knowledge, the first in Tunisia, and one of a few studies focusing on the evaluation of QOL and work productivity and activity impairment among patients with allergic OR. Although some limitations were observed, such as the bias of self-administrated questionnaires with possible misunderstanding of questions or overestimation of scores, the use of a specific questionnaire for the evaluation of QOL and Work impairment strengthen our results. Existence of an Arabic version of the Mini-RQLQ enhanced the comprehension of questions with lesser possible comprehension errors. WPAI-AS have been already validated with various allergic diseases including rhinitis (26).

Textile and clothing companies are essentially located in the Tunisian central region explaining the predominance of patients working in this sector and diagnosed with allergic OR.

Patients with allergic OR do not only complain of clinical problems related to their symptomatology, but also of problems related to their occupation, such as inability to continue work, indication of allergen eviction and possible job loss. These professional changes can also interact with QOL. In the literature, several studies have evaluated the QOL in patients with occupational asthma (27–29) but only few studies explored QOL in patients with OR.

In our study, female workers had more impaired QOL scores. Housekeeping activities, which were evaluated by the questionnaire may explain a part of this findings. Women are generally cumulating efforts at work and at home and are exposed to household products that can aggravate rhinitis symptoms, impacting QOL.

These findings were similar to those reported by Shariat et al*.* (16) conducted in 2011 among 110 patients with non-occupational allergic rhinitis. Although we found an association between impairment of QOL, textile and clothing sector and professional seniority, similar results were not noted in the literature. Clothing sector is very developed in our country especially in the central region where most companies are set up. Workers in this sector are generally women with low educational level and from disadvantaged class accepting to stay at work despite their functional complaints. Maintaining exposure is a major possible reason for aggravation of symptoms and, by the same way, decline of perceived QOL.

Other studies mainly focused on the impact of exposure cessation on QOL. Gerth van Wijk et al. concluded that work cessation had beneficial on improving the QOL of patients with occupational rhinitis (30). Power et al. found that among their study population of 29 patients allergic to latex, 90% noted that the total suppression of the allergen resulted in disappearance of the effects of nasal and ocular symptoms on their QOL (31).

Using the WPAI questionnaire, several other studies showed an impairment of work productivity and activity among patients with allergic rhinitis. Studies of Bousquet et al. (14), Small et al. (18) and De la Hoz Caballer et al. (20), showed an absenteeism ranging from 0 to 4.6%, a presenteeism ranging from 18.1 to 23.5%, a work productivity loss from 18.9 to 26.6% and an overall activity impairment ranging from 20 to 27.8%. Except for absenteeism, our results showed more important impairment of WPAI than these three studies. Absenteeism and presenteeism varies from one country to another due to cultural, socioeconomic, and health insurance factors. In our study, poorly qualified blue collars were the most represented category. Job insecurity because of possible loss of job is a threat for these workers who maintain their activity and avoid medical leave. Maintaining activity while symptoms are important or severe can explain an increase of presenteeism and a decline of productivity.

Compared to other diseases, allergic rhinitis seemed to have more negative impact on work productivity and activity than hypertension and diabetes and only depression caused more impairment than allergic rhinitis in the study of De la Hoz Caballer(20).

Our study showed associations between QOL and work productivity among patients with allergic OR. Even if absenteeism rates seem to be moderate, work productivity is clearly reduced because of important presenteeism percentages.

Identification of factors such as age, gender and QOL impairment could help to identify workers with higher risk of productivity and activity impairment. A rigorous application of preventive measures and a medical control of the disease should reduce the burden of allergic occupational rhinitis and also improve QOL and work productivity.

## CONCLUSION

Allergic OR impairs QOL and work productivity. Although it doesn’t seem to be associated with an important absenteeism, work productivity is reduced by an important rate of presenteeism. QOL and work productivity seem to interact significantly.

Only few studies were conducted to evaluate the impact of allergic OR on QOL and/or work productivity. This subject needs to be more explored for many reasons: the important incidence of the disease in various occupational sectors, its negative repercussions on QOL and on productivity, and the expected positive contribution of prevention and treatment in the improvement of social and economic aspects associated with occupational OR.

## References

[1] Dykewicz MS Fineman S Skoner DP Nicklas R Lee R Blessing-Moore J Diagnosis and management of rhinitis: complete guidelines of the Joint Task Force on Practice Parameters in Allergy, Asthma and Immunology. American Academy of Allergy, Asthma, and Immunology. Ann Allergy Asthma Immunol 1998;81(5 Pt 2):478–518. 9860027 10.1016/s1081-1206(10)63155-9

[2] Moscato G Vandenplas O Van Wijk RG Malo JL Perfetti L Quirce S EAACI position paper on occupational rhinitis. Respir Res 2009;10:16. 19257881 10.1186/1465-9921-10-16 PMC2654869

[3] Garnier R Villa A Chataigner D Rhinites professionnelles. Rev Mal Respir 2007;24 (2): 205–220. 17347607 10.1016/s0761-8425(07)91043-8

[4] Brozek JL Bousquet J Baena-Cagnani CE Bonini S Canonica GW Casale TB Allergic Rhinitis and its Impact on Asthma (ARIA) guidelines: 2010 revision. J Allergy Clin Immunol 2010;126(3):466–76. 20816182 10.1016/j.jaci.2010.06.047

[5] Château-Waquet D La rhinite allergique. Décision thérapeutique en médecine générale 2006;31:8–13.

[6] Didier A Chanal I Klossek JM Mathieu J Bousquet J La rhinite allergique: le point de vue du patient. Revue francaise d’allergologie et d’immunologie clinique 1999;39(3):171–85.

[7] Mallol J Crane J von Mutius E Odhiambo J Keil U Stewart A ISAAC Phase Three Study Group The International Study of Asthma and Allergies in Childhood (ISAAC) Phase Three: a global synthesis. Allergol Immunopathol (Madr) 2013;41(2):73–85. 22771150 10.1016/j.aller.2012.03.001

[8] Dürr C Heimgartner S Gehrig R Caversaccio M Helbling A Allergie aux pollens: aspects cliniques. Première partie. InForum Médical Suisse 2008; 8(14): 253–7.

[9] Aranđelović M Stanković I Jovanović JM Borisov S Stanković S Allergic rhinitis: Possible occupational disease: Criteria suggestion. Acta Facultatis Medicae Naissensis 2004;21(2):65–71.

[10] Magroun I Daly L Hamdouni M Maladies Allergiques respiratoires d’origine Professionnelle Aspects cliniques et état des lieux en Tunisie. SST 2014;66:2–13.

[11] Beaudoin E Kanny G Jankowski R Stringini R Moneret-Vautrin DA Les rhinites allergiques professionnelles. Annales d’oto-laryngologie et de chirurgie cervico-faciale. Elsevier Masson; 1994;111(3):. 115–9. 7840482

[12] Sublett JW Bernstein DI Occupational rhinitis. Immunol Allergy Clin North Am 2011;31(4):787–96, vii. 21978857 10.1016/j.iac.2011.07.007

[13] Leimgruber A Spertini F Allergic rhinoconjunctivitis and immunotherapy: an update. Rev Med Suisse 2009;5(186):88–93. 19238924

[14] Bousquet J Neukirch F Bousquet PJ Gehano P Klossek JM Le Gal M Severity and impairment of allergic rhinitis in patients consulting in primary care. J Allergy Clin Immunol 2006;117(1):158–62. 16387600 10.1016/j.jaci.2005.09.047

[15] Meltzer EO Nathan RA Seiner JC Storms W Quality of life and rhinitic symptoms: results of a nationwide survey with the SF-36 and RQLQ questionnaires. Journal of allergy and clinical immunology 1997;99(6):S815–9.

[16] Shariat M Pourpak Z Khalesi M Kazemnejad A Sharifi L Souzanchi G Quality of life in the Iranian adults with allergic rhinitis. Iran J Allergy Asthma Immunol 2012;11(4):324–8. 23264409

[17] Canonica GW Bousquet J Mullol J Scadding GK Virchow JC A survey of the burden of allergic rhinitis in Europe. Allergy 2007;62 Suppl 85:17–25. 10.1111/j.1398-9995.2007.01549.x 17927674

[18] Small M Piercy J Demoly P Marsden H Burden of illness and quality of life in patients being treated for seasonal allergic rhinitis: a cohort survey. Clin Transl Allergy 2013 9;3(1):33. 24107462 10.1186/2045-7022-3-33 PMC3852977

[19] Leynaert B Neukirch C Liard R Bousquet J Neukirch F Quality of life in allergic rhinitis and asthma. A population-based study of young adults. Am J Respir Crit Care Med 2000;162(4 Pt 1):1391–6. 11029350 10.1164/ajrccm.162.4.9912033

[20] de la Hoz Caballer B Rodríguez M Fraj J Cerecedo I Antolín-Amérigo D Colás C Allergic rhinitis and its impact on work productivity in primary care practice and a comparison with other common diseases: the Cross-sectional study to evAluate work Productivity in allergic Rhinitis compared with other common dIseases (CAPRI) study. Am J Rhinol Allergy 2012;26(5):390–4. 23168153 10.2500/ajra.2012.26.3799 PMC3904041

[21] Juniper EF Guyatt GH Development and testing of a new measure of health status for clinical trials in rhinoconjunctivitis. Clinical & Experimental Allergy 1991;21(1):77–83. 2021881 10.1111/j.1365-2222.1991.tb00807.x

[22] Juniper EF How to develop and validate a new health-related quality of life instrument. Quality of life and pharamacoeconomics in clinical trials 1996:49–56.

[23] AbuRuz SM Bulatova NR Tawalbeh MI Development and validation of the Arabic allergic rhinitis quality of life questionnaire. Saudi Med J 2009;30(12):1577–83. 19936424

[24] Baur X Degens P Weber K Occupational obstructive airway diseases in Germany. Am J Ind Med 1998;33(5):454–62. 9557168 10.1002/(sici)1097-0274(199805)33:5<454::aid-ajim4>3.0.co;2-s

[25] Baur X Arbeitsmedizinische Aspekte der nasalen Erkrankungen. Laryngo-Rhino-Otologie 1998;77(04):191–5. 9592751 10.1055/s-2007-996959

[26] Reilly MC Tanner A Meltzer EO Work, classroom and activity impairment instruments. Clinical drug investigation 1996;11(5):278–88.

[27] Malo JL Boulet LP Dewitte JD Cartier A L’Archevêque J Côté J Quality of life of subjects with occupational asthma. J Allergy Clin Immunol 1993;91(6):1121–7. 8509574 10.1016/0091-6749(93)90313-5

[28] Karvala K Uitti J Luukkonen R Nordman H Quality of life of patients with asthma related to damp and moldy work environments. Scand J Work Environ Health 2013;39(1):96–105. 22407188 10.5271/sjweh.3289

[29] Sauni R Oksa P Vattulainen K Uitti J Palmroos P Roto P The effects of asthma on the quality of life and employment of construction workers. Occup Med (Lond) 2001;51(3):163–7. 11385120 10.1093/occmed/51.3.163

[30] Gerth van Wijk R Patiwael JA de Jong NW de Groot H Burdorf A Occupational rhinitis in bell pepper greenhouse workers: determinants of leaving work and the effects of subsequent allergen avoidance on health-related quality of life. Allergy 2011;66(7):903–8. 21303376 10.1111/j.1398-9995.2011.02556.x

[31] Power S Gallagher J Meaney S Quality of life in health care workers with latex allergy. Occup Med (Lond) 2010;60(1):62–5. 19901008 10.1093/occmed/kqp156

